# 2-Substituted Aniline as a Simple Scaffold for LuxR-Regulated QS Modulation

**DOI:** 10.3390/molecules22122090

**Published:** 2017-11-29

**Authors:** Sizhe Li, Julien Wawrzyniak, Yves Queneau, Laurent Soulère

**Affiliations:** 1 Univ Lyon, INSA LYON, Université Lyon 1, CPE Lyon, UMR 5246, CNRS, ICBMS, Bât. J. Verne, 20 Avenue A. Einstein, F-69621 Villeurbanne, France; sizhe.li@insa-lyon.fr; 2 Univ Lyon, INSA Lyon, Université Lyon 1, UMR 5240, CNRS, MAP, 10 rue Raphaël Dubois, F-69622 Villeurbanne, France; julien.wzk@gmail.com

**Keywords:** quorum sensing, antagonists, 2-nitroaniline scaffold, molecular docking

## Abstract

The ability of the 2-substituted aniline motif to serve as a scaffold for designing potential LuxR-regulated quorum sensing (QS) modulators has been investigated, using docking experiments and biological evaluation of a series of 15 specially synthesized compounds. Aniline, 2-acetyl-aniline and 2-nitroaniline were considered, as well as their *N*-acylated derivatives. Docking experiments showed that the 2-substituted aniline motif fits within the LuxR binding site at the place of the lactone moiety of AHL, and the biological evaluation revealed QS antagonisitic activity for several compounds, validating the hypothesis that this scaffold acts on QS. Structure activity relationships are discussed regarding interactions with the key residues of the LuxR binding site, showing significant variations in the H-bonding pattern.

## 1. Introduction

**Figure 1:**
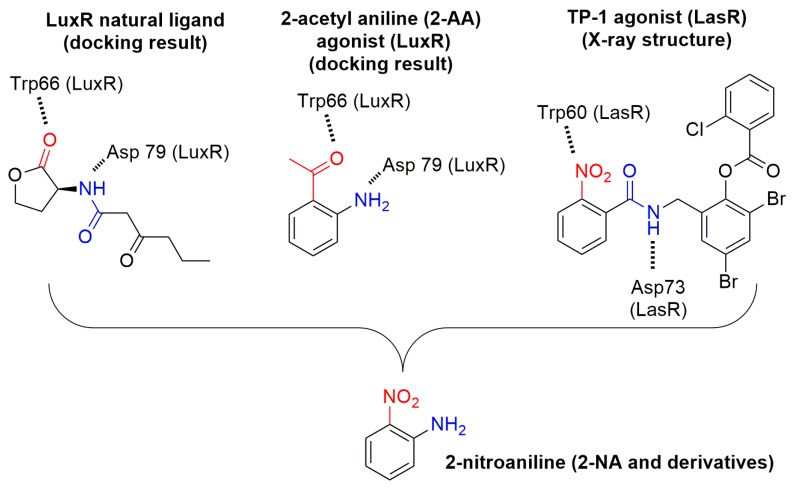
Structure-based design of 2-nitroaniline (**2-NA**) and derivatives (**2-NA**).

Bacterial quorum sensing (QS) is a communication system based on the biosynthesis of small diffusible molecules, called autoinducers, able to interact with proteins, in particular transcriptional factors [1]. This communication system, and, more specifically, its quenching using small molecules to fight bacterial virulence, has been extensively studied [2,3,4,5,6,7]. In Pseudomonas, to date, three QS systems have been identified [8]. Two of them are regulated by acyl homoserine lactones (AHLs), namely *N*-3-oxo-dodecanoyl-homoserine lactone and *N*-butanoyl-homoserine lactone, and they interact, respectively, with LasR and RhlR. Another system, called PQS (Pseudomonas quinolone signal), is regulated via quinolone derivatives. These compounds are biosynthesized from anthranilic acid, yielding HHQ [9]. Recently, a secondary metabolite from this biosynthetic pathway, namely 2-aminoacetophenone (**2-AA**), has been shown, by Kviatkovski and co-workers [10], to activate LuxR. In their work, they also used molecular modeling to demonstrate that **2-AA** replaces the lactone, within the ligand binding site of a LuxR model, with hydrogen bonds between the C=O and Trp66 and between the amine and Asp79. The 2-nitrobenzamide derivative TP-1 [11] (Figure 1) and other 2-nitrobenzamide derivatives have also been shown to be LasR modulators [12]. The X-ray-analyzed structure of the TP-1/LasR complex [11] shows that the 2-nitrobenzamide moiety, like **2-AA**, is positioned at the same place as the lactone of AHL, with hydrogen bonding between the nitro group and Trp60 and also between the NH of the amide function and Asp73. These observations led us to investigate whether other 2-substituted anilines, in particular 2-nitroaniline (**2-NA**), act as new simple scaffolds for QS modulation. We report here the results of our study, using docking and biological screening, on a series of 2-acetyl-aniline and 2-nitroaniline compounds, as well as their *N*-acylated derivatives, as potential QS modulators.

## 2. Results and Discussion

**Figure 2:**
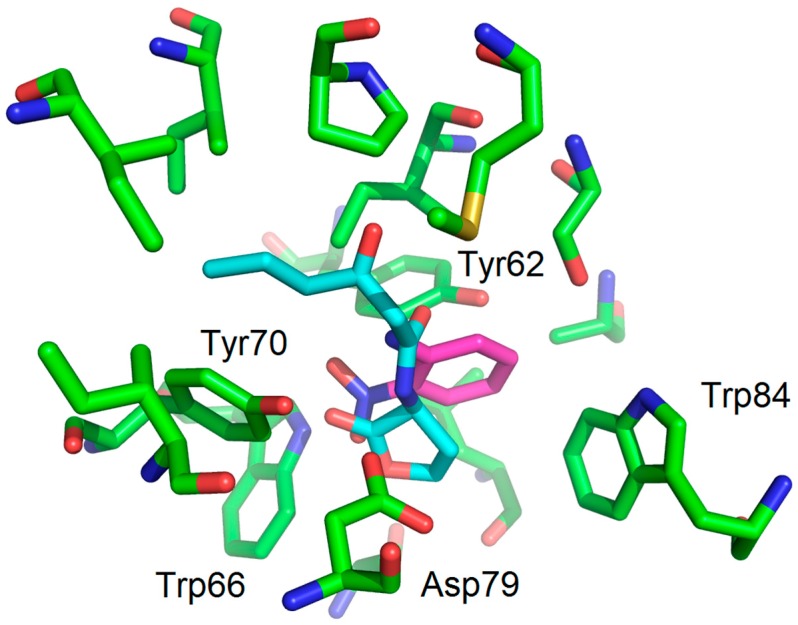
Proposed binding mode obtained as a result of docking experiments of **2-NA** (magenta) within the LuxR model binding site. OHHL is also represented, in cyan. Docking experiments were performed using ArgusLab as software with a genetic algorithm as the docking engine (GADock) (see experimental section).

Flexible docking investigations [13,14] of *N*-3-oxo-hexanoyl-homoserine (OHHL) [15] and 2-nitroaniline using a LuxR model described in 2007 by Soulère and co-workers [16] showed that 2-nitroaniline is mimicking the lactone ring, notably forming hydrogen bonds between the nitro group (replacing the ester function of OHHL) and Trp66 (Figure 2).

**Figure 3:**
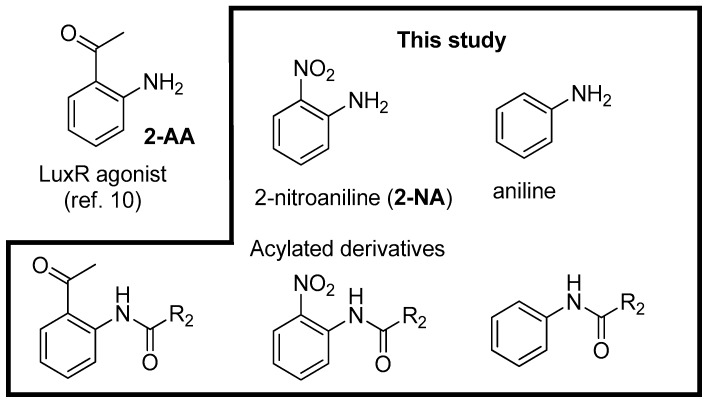
Structure of substituted anilines and their acylated derivatives.

In keeping with the same structural analogy, in which the amine function of **2-NA** or **2-AA** (see supplementary data) and the NH group of the amide function of OHHL appear in similar positions within the binding site, we included in this study their *N*-acylated derivatives, thus mimicking the amide side chain of AHLs. For comparison, we also investigated acyl anilines to examine the effect of this substituent in position 2 (Figure 3).

**Figure 4:**
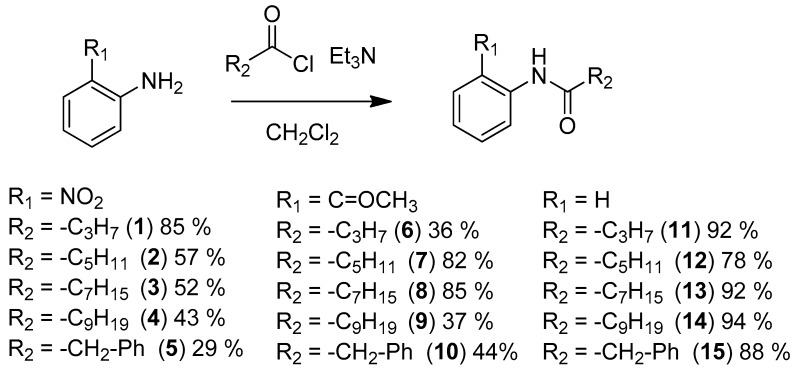
Synthesis and structure of acyl aniline derivatives as LuxR modulators.

All compounds were easily prepared from **2-NA** [17], **2-AA** or aniline by direct acylation with the corresponding acyl chloride (Figure 4). For compounds **5**, **10**, and **15**, the acyl chloride was synthesized using oxalyl chloride from 4-phenylbutyric acid with catalytic dimethylformamide.

**Figure 5:**
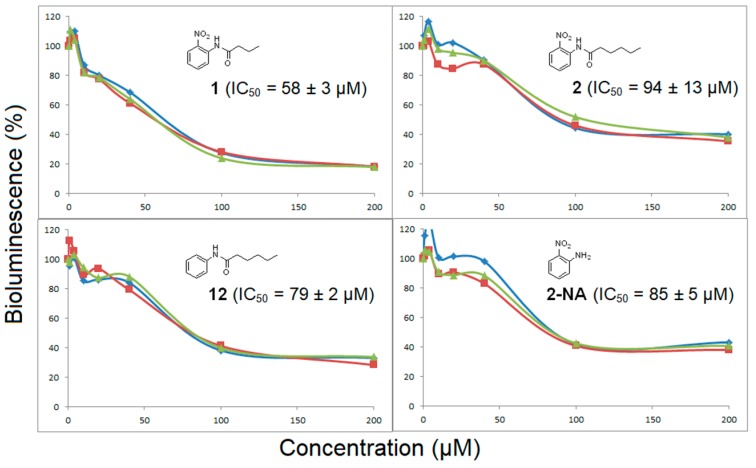
Antagonistic activity of **2-NA,** and acyl 2-nitroaniline derivatives **1**–**2** and acyl aniline **12**. Other derivatives were inactive (not shown). Bioluminescence was induced with 200 nM of 3-oxo-C6-HSL. The experiments were carried out in triplicate shown in different colors.

For the biological evaluation [18], all compounds were evaluated as agonists for their ability to induce bioluminescence in the *V. fischeri* QS system with a recombinant *Escherichia coli* biosensor. This biosensor strain produces luminescence with exogenously-provided AHL. None of them were found to be agonists, i.e., they did not induce bioluminescence. Compounds were also tested, at various concentrations, as antagonists in competition with 200 nM of 3-oxo-C6-HSL (Figure 5).

**Figure 6:**
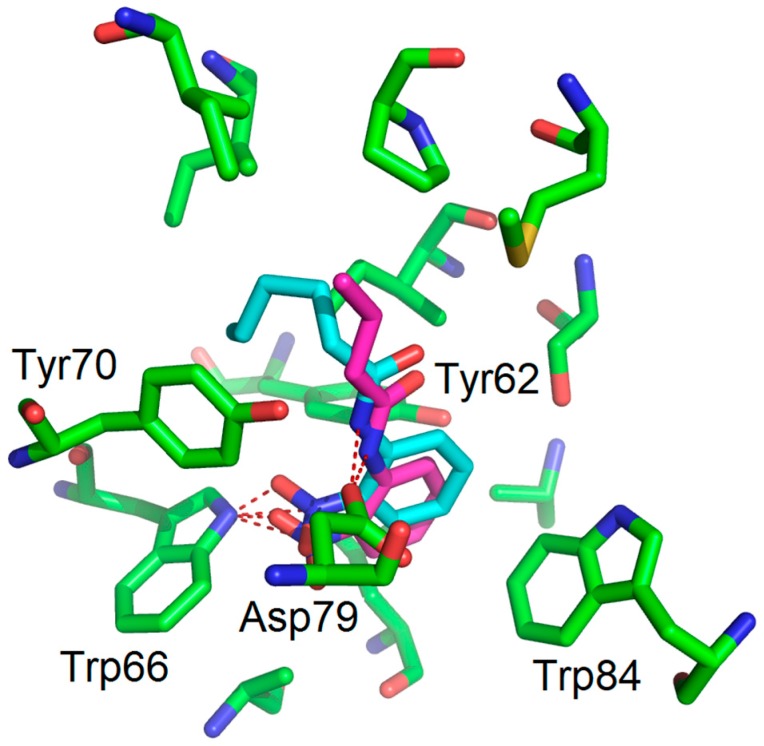
Proposed binding mode obtained as a result of the docking experiment with compound **1** (magenta) and **2** (cyan) showing that the phenyl group and the alkyl chain fit within the LuxR model binding site.

Interestingly, these assays revealed that **2-NA**, *N*-(2-nitrophenyl)butanamide (**1**) and *N*-(2-nitrophenyl)hexanamide (**2**) all displayed antagonist activity whereas other acyl derivatives, **3**–**10**, were totally inactive. Based on the LuxR-QS system, compounds **1** and **2** exhibited IC_50_ values of 58 µM and 94 µM, respectively, comparable with many other compounds described in the literature, thus validating our rational approach [5]. For acyl nitroaniline **3**–**5**, with longer acyl chains (C8 and C10) or with a terminal phenyl group, their inactivity can be explained by an inappropriate size of the substituent R_2_, like what was observed for other structurally close AHL analogues with long chains [5]. As shown in Figure 6, docking experiments, with compounds **1** and **2** (nitro-C4 and nitro-C6) within the LuxR model binding site, revealed a satisfactory fit of these compounds, with a binding mode of hydrogen bonds between Trp66 and the nitro function and between Asp79 and the NH group. The aromatic part of the compound fits well and replaces the lactone moiety. The difference in activity for compounds **1** and **2** may be due to the change in the orientation of the alkyl chain.

**Figure 7:**
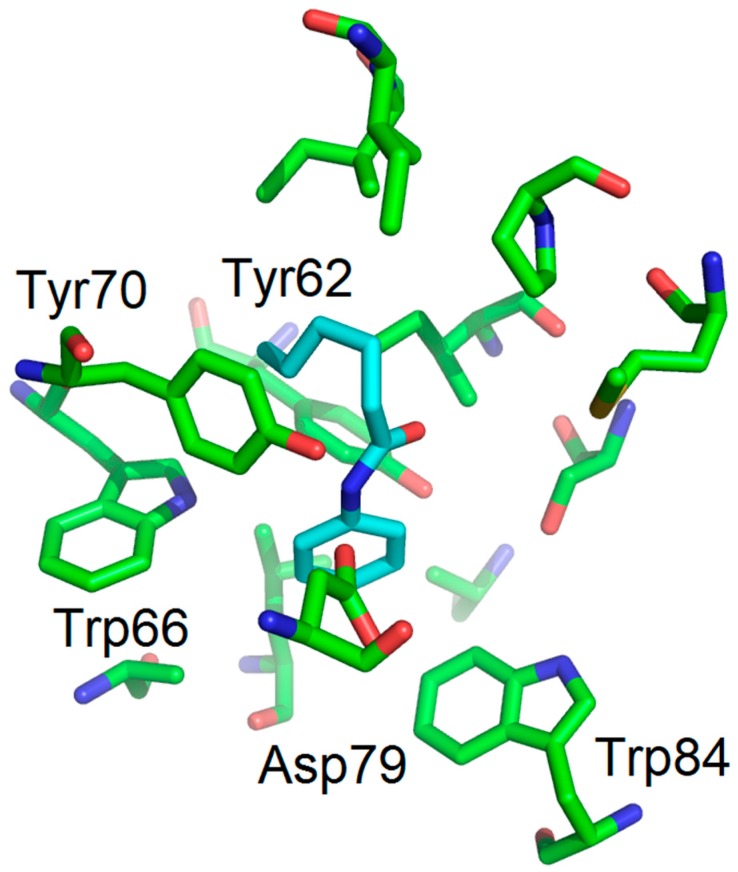
The binding mode (molecular docking) of compound **12** (cyan) shows a good fit, through hydrogen bonding, with Asp79 (NH).

A comparison (biological evaluation and docking experiments) of 2-unsubstituted anilines was then performed. As already reported with LuxR for compound **14** [19], compounds **13** and **15** were found to be inactive. Interestingly, the hexanamide **12** showed significant antagonist activity, with an IC_50_ value of 79 µM, in the same range as *N*-(2-nitrophenyl)hexanamide (**2**). However, to our surprise, further shortening of the chain to C4 (compound **11**) led to a total loss of activity, whereas the corresponding nitrated counterpart was found to be the most active compound in this study. This shows that the two families of compounds behave differently, with a crucial nitro-substituent which anchors the molecules within the binding site, via a hydrogen bond with Trp66, and a less important chain length effect rendering both the C4 and the C6 derivatives active. On the other hand, for the unsubstituted aniline series, only one very specific derivative, the C6 amide, displays sufficient favorable interaction and sterical balance. This hypothesis is supported by docking experiments showing only one favorable binding mode for hexanoylaniline **12** within the LuxR binding site (Figure 7) whereas several binding modes, all unfavorable, were found for compound **11** within the LuxR binding site.

We next examined the H-bond interactions involving the important and conserved residues of the binding site (Trp66, Asp79 and Tyr62) for **2-AA**, **2-NA**, and compounds **1**–**2**, **6**–**7** and **11**–**12**, with the appropriate R_2_ substituents (Table 1).

The autoinducer OHHL displays three hydrogen bonds with Trp66, Asp79, and Tyr62. The agonist **2-AA** displays two hydrogen bonds with the same residues (none with Tyr62 due to the absence of the carboxamide function). In contrast, **2-NA**, which shows antagonist activity, interacts only with Trp66, but in this case through two hydrogen bonds between Trp66 and the nitro group, and it does not interact with Asp79. The binding modes for **2-AA** (agonist) and **2-NA** (antagonist) are, therefore, significantly different with respect to the H-bond interactions. The acylated nitroanilines **1** and **2** display two hydrogen bonds, one with Trp66 and one with Asp79, whereas the simpler hexanoylaniline **12** shows only one hydrogen bond with Asp79. Thus, antagonists **1**, **2** and **12** all display hydrogen bonds with Asp79 and Trp66 (if possible) but not with Tyr62. The combination of satisfactory overall binding and the absence of any interaction with Tyr62 appear to be a common feature among compounds showing antagonistic activity. For the inactive compound **11**, docking experiments showed several binding modes due to the size of this compound and the absence of the nitro group. For compounds **6** and **7**, they adopt a binding mode with the same hydrogen bond network as OHHL, including the interaction with Tyr62.

## 3. Materials and Methods

### 3.1. Binding Mode Studies

**Figure 8:**
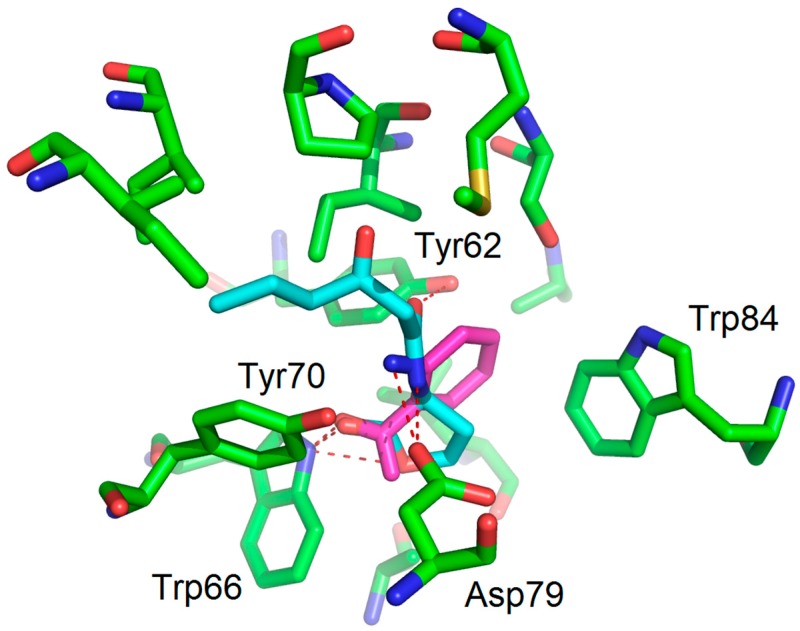
The binding mode (molecular docking) of **2-AA** (magenta) within the LuxR model binding site. OHHL is also represented, in cyan.

Docking experiments were performed with the docking module of the ArgusLab software 4.0.1 (Planetaria Software LLC.: Seattle, WA, USA) [14]. The protein model of LuxR [16] was built using SWISS-MODEL [20] with ClustalW [21]. The binding mode of OHHL was obtained using the method described by Estephane and co-workers [15]. Docking experiments were performed with the following parameters: Docking box: X = Y = Z = 15 Å, ligand option: flexible; calculation type: Dock; Docking engine: GADock (Genetic Algorithm) [13]; Genetic algorithm dock settings: default advanced parameters; hydrogen bonds were assigned within a distance of 3 Å. Figures were generated using PyMOL. The binding mode with hydrogen bonds of **2-AA** (magenta) is shown in the following figure (Figure 8) and it is in keeping with that obtained by Kviatkovski and co-workers [10].

### 3.2. Biological Evaluation

Compounds were evaluated for their ability to induce bioluminescence (agonistic activity) in the *V. fischeri* QS system. For this purpose, we used the recombinant *Escherichia coli* biosensor strain NM522 containing the plasmid pSB401. In this plasmid, the luxR and the luxI promoters from *V. fischeri* are associated with the lux structural operon (luxCDABE) from *Photorhabdus luminescens* [18,22]. This biosensor strain produces luminescence with exogenously provided AHL. The activity was measured using a microtiter plate format (Fisher Scientific, Waltham, MA, USA). A competition assay (antagonistic activity) was also performed in the presence of 200 nM of 3-oxo-C6-HSL and with simultaneously added analogs. The experiments were carried out in triplicate with a maximum of 2% DMSO. A negative control was performed in the absence of 3-oxo-C6-HSL and this gave the basal level of bioluminescence in the absence of an inducer. A positive control was performed in the presence of 3-oxo-C6-HSL only.

### 3.3. Synthesis

Chemical reagents were purchased from Sigma Aldrich (St. Louis, MO, USA). Flash chromatography was performed using 60 M silica gel. All reactions were monitored with thin-layer chromatography (TLC) carried out on Merck aluminum silica gel 60-F254 (Darmstadt, Germany) using UV light and a KMnO_4_ solution as the stain. ^1^H nuclear magnetic resonance (NMR) spectra were obtained on Bruker ALS300 and DRX300 spectrometers (Billerica, MA, USA).

#### 3.3.1. General Procedure for the Synthesis of *N*-(2-Nitrophenyl) Amide

To a solution of 2-nitroaniline (1.0 eq), in 10 mL anhydrous dichloromethane, was added triethylamine (2.5 eq) and acyl chloride (2.5 eq: butyryl chloride, hexanoyl chloride, octanoyl chloride, decanoyl chloride, 4-phenylbutanoyl chloride was prepared from 4-phenylbutanoic acid and oxalyl chloride with catalytic DMF) at 0 °C. The reaction mixture was left to warm to room temperature, with stirring, for 16 h. The mixture was diluted in 30 mL dichloromethane and washed with 40 mL 2 M HCl, 40 mL 1 M NaOH and 40 mL of brine. The organic layer was dried over anhydrous Na_2_SO_4_, evaporated and then the residue was purified by flash chromatography column to give the required products (29% to 85% yield).

#### 3.3.2. General Procedure for the Synthesis of *N*-phenyl Amide Compounds and *N*-(2-acetylphenyl) Amide Compounds

To a solution of aniline or 2-Aminoacetophenone (1.0 eq), in 10 mL anhydrous dichloromethane, was added triethylamine (1.1 eq) and acyl chloride (1.1 eq: butyryl chloride, hexanoyl chloride, octanoyl chloride, decanoyl chloride, 4-phenylbutanoyl chloride was prepared from 4-phenylbutanoic acid and oxalyl chloride with catalytic DMF) at 0 °C. The reaction mixture was left to warm to room temperature, with stirring, for 16 h. The mixture was diluted in 30 mL dichloromethane and washed with 40 mL 1 M HCl, 40 mL sat. NaHCO_3_ and 40 mL brine. The organic layer was dried over anhydrous Na_2_SO_4_, evaporated and then the residue was purified by flash chromatography column to give the required products (36% to 94% yields).

*N-(2-Nitrophenyl)butyramide* (**1**) see reference [17].

*N-(2-Nitrophenyl)hexanamide* (**2**) see reference [17].

*N-(2-Nitrophenyl)octanamide* (**3**) see reference [17].

*N-(2-Nitrophenyl)decanamide* (**4**). Purification: pentane/diethyl ether = 10/1; yellow solid (43%); ^1^H-NMR (300 MHz, Chloroform-*d*) δ 10.30 (s, 1H, NH), 8.74 (dd, *J* = 8.6, 1.4 Hz, 1H, Ph-H), 8.15 (dd, *J* = 8.5, 1.6 Hz, 1H, Ph-H), 7.58 (ddd, *J* = 8.8, 7.2, 1.6 Hz, 1H, Ph-H), 7.10 (ddd, *J* = 8.5, 7.2, 1.4 Hz, 1H, Ph-H), 2.42 (t, *J* = 7.5 Hz, 2H, COCH_2_), 1.69 (p, *J* = 7.5 Hz, 2H, CH_2_), 1.35–1.11 (m, 12H, 6CH_2_), 0.81 (t, *J* = 7.4 Hz, 3H, CH_3_). ^13^C-NMR (75 MHz, Chloroform-*d*) δ 172.4, 136.3, 136.1, 135.2, 125.9, 123.2, 122.3, 38.9, 32.0, 29.5, 29.43, 29.37, 29.26, 25.5, 22.8, 14.2. HR-MS (ESI positive mode) calculated for C_16_H_25_N_2_O_3_^+^: 293.1860. Found: 293.1846.

*N-(2-Nitrophenyl)-4-phenylbutanamide* (**5**). Purification: pentane/AcOEt =15/1; yellow liquid (29%); ^1^H-NMR (300 MHz, Chloroform-*d*) δ 10.38 (d, *J* = 5.5 Hz, 1H, NH), 8.83 (d, *J* = 8.3 Hz, 1H, Ph-H), 8.46–8.15 (m, 1H, Ph-H), 7.85–7.49 (m, 1H, Ph-H), 7.44–7.02 (m, 6H, Ph-H), 2.77 (t, *J* = 7.3 Hz, 2H, PhCH_2_), 2.55 (t, *J* = 7.3 Hz, 2H, COCH_2_), 2.16 (q, *J* = 7.4 Hz, 2H, CH_2_). ^13^C-NMR (75 MHz, Chloroform-*d*) δ 171.0, 140.2, 135.4, 135.1, 134.0, 127.6, 125.3, 124.9, 122.3, 121.3, 36.9, 34.2, 25.8. HR-MS (ESI positive mode) calculated for C_16_H_17_N_2_O_3_^+^: 285.1234. Found: 285.1231.

*N-(2-Acetylphenyl)butyramide* (**6**). Purification: pentane/AcOEt =15/1; white solid (36%); ^1^H-NMR (300 MHz, Chloroform-*d*) δ 11.71 (s, 1H, NH), 8.95–8.61 (m, 1H, Ph-H), 7.97–7.79 (m, 1H, Ph-H), 7.55 (dddd, *J* = 8.5, 7.3, 1.6, 0.5 Hz, 1H, Ph-H), 7.10 (ddd, *J* = 8.0, 7.3, 1.2 Hz, 1H, Ph-H), 2.67 (s, 3H, COCH_3_), 2.41 (t, *J* = 7.5 Hz, 2H, COCH_2_), 1.92–1.66 (m, 2H, CH_2_), 1.01 (t, *J* = 7.4 Hz, 3H, CH_3_). ^13^C-NMR (75 MHz, Chloroform-*d*) δ 202.9, 172.7, 141.2, 135.3, 131.7, 122.3, 121.7, 120.8, 40.8, 28.7, 19.1, 13.9. HR-MS (ESI positive mode) calculated for C_12_H_15_NNaO_2_^+^: 228.0995. Found: 228.0985.

*N-(2-Acetylphenyl)hexanamide* (**7**) see reference [23].

*N-(2-Acetylphenyl)octanamide* (**8**). Purification: pentane/AcOEt =15/1; colorless oil (85%); ^1^H-NMR (300 MHz, Chloroform-*d*) δ 11.70 (s, 1H, NH), 8.77 (dd, *J* = 8.5, 1.2 Hz, 1H, Ph-H), 7.89 (dd, *J* = 8.0, 1.6 Hz, 1H, Ph-H), 7.54 (ddd, *J* = 8.5, 7.2, 1.6 Hz, 1H, Ph-H), 7.10 (ddd, *J* = 8.0, 7.3, 1.2 Hz, 1H, Ph-H), 2.66 (s, 3H, COCH_3_), 2.43 (t, *J* = 7.5 Hz, 2H, COCH_2_), 1.83–1.62 (m, 2H, CH_2_), 1.44–1.16 (m, 8H, 4CH_2_), 0.96–0.72 (t, *J* = 7.4 Hz, 3H, CH_3_). ^13^C-NMR (75 MHz, Chloroform-*d*) δ 202.9, 172.9, 141.3, 135.3, 131.7, 122.3, 121.8, 120.9, 38.9, 31.8, 29.3, 29.1, 28.7, 25.7, 22.7, 14.2. HR-MS (ESI positive mode) calculated for C_16_H_24_NO_2_^+^: 262.1802. Found: 262.1793.

*N-(2-Acetylphenyl)decanamide* (**9**) see reference [24].

*N-(2-Acetylphenyl)-4-phenylbutanamide* (**10**). Purification: pentane/AcOEt =13/1; colorless oil (44%); ^1^H-NMR (300 MHz, Chloroform-*d*) δ 11.75 (s, 1H, NH), 8.80 (dd, *J* = 8.5, 1.2 Hz, 1H, Ph-H), 7.92 (dd, *J* = 8.0, 1.6 Hz, 1H, Ph-H), 7.70–7.50 (m, 1H, Ph-H), 7.41–7.06 (m, 6H, Ph-H), 2.75 (t, *J* = 7.4 Hz, 2H, PhCH_2_), 2.69 (s, 3H, COCH_3_), 2.50 (t, *J* = 7.5 Hz, 2H, COCH_2_), 2.20–2.05 (m, 2H, CH_2_). ^13^C-NMR (75 MHz, Chloroform-*d*) δ 202.9, 172.4, 141.6, 141.2, 135.3, 131.7, 128.6, 128.5, 126.1, 122.4, 121.8, 120.9, 38.1, 35.3, 28.7, 27.1. HR-MS (ESI positive mode) calculated for C_18_H_20_NO_2_^+^: 282.1489. Found: 282.1483.

*N-Phenylbutyramide* (**11**) see reference [25].

*N-Phenylhexanamide* (**12**) see reference [26].

*N-Phenyloctanamide* (**13**) see reference [27].

*N-Phenyldecanamide* (**14**) see reference [28].

*N,4-Diphenylbutanamide* (**15**) see reference [29].

## 4. Conclusions

In our search for a novel scaffold that is active on LuxR-regulated QS and based on a benzenic backbone, we designed an original combination of substituents, notably the nitro and acylated amine groups. Docking experiments demonstrated that this scaffold replaces exactly the lactone moiety of AHL within the binding site of LuxR. However, variations were observed in the hydrogen network between the ligands and some key amino acids of the binding site, with some significant results revealed by the biological evaluation. In particular, the absence of the hydrogen bond with Tyr62 appears to be essential for antagonistic activity in this new family of aniline and nitroaniline QS inhibitors. The design and the docking experiments were validated by biological assays showing significant inhibitory activity, notably for *N*-(2-nitrophenyl)butyramide (**1**). Thus, 2-nitro aniline, a very simple and versatile scaffold, has proved to be a valuable basis for designing novel QS-active candidates.

## Figures and Tables

**Table 1:** Occurrence and distances for hydrogen bonds between Trp66, Asp79, and Tyr 62 and the main chemical functions of studied compounds with distances (Å) ^a,b^.

Compounds	Trp66	Asp79	Tyr62
OHHL (natural ligand)	+2.34 (C=O lactone) ^a^	+3.01 (NH amide)	+3.01 (C=O amide)
**2-AA (agonist)**	+2.20 (C=O)	+3.03 (NH_2_)	No function
**2-NA (antagonist)**	+2.96 and 2.37 (NO_2_)	-NH_2_ too far	No function
**1 (antagonist)**	+2.47 (NO_2_)	+3.00 (NH amide)	-C=O too far
**2 (antagonist)**	+2.20 (NO_2_)	+3.01 (NH amide)	-C=O too far
**6** (No activity)	+1.99 (C=OCH_3_)	+3.10 (NH amide)	+2.97 (C=O amide)
**7** (No activity)	+2.01 (C=OCH_3_)	+2.98 (NH amide)	+2.94 (C=O amide)
**11** (No activity)	-	-	-
**12 (antagonist)**	No function	+3.04 (NH amide)	-C=O too far

^a^ + indicates a possible H-bond; ^b^ the function implicated is indicated in brackets.

## Supplementary Materials

Supplementary materials are available online. ^1^H-NMR spectra.
