# Supplementary material for: 2-Substituted Aniline as a Simple Scaffold for LuxR-Regulated QS Modulation
Source: Molecules. 2017 Nov 29;22(12):2090. doi: 10.3390/molecules22122090 (PMC6149922; doi:10.3390/molecules22122090)
Supplement: Supplementary file 1 [file molecules-22-02090-s001.pdf]

2-Substituted aniline as simple scaffold for LuxR-regulated QS modulation

Si-Zhe Li,[1] Julien Wawrzyniak,[2] Yves Queneau,[* 1] and Laurent Soulère[*1]

[1] Univ Lyon, INSA Lyon, Université Lyon 1, CPE Lyon, UMR 5246, CNRS, ICBMS, Institut de Chimie et de Biochimie Moléculaires et Supramoléculaires, Bât. J. Verne, 20 avenue A. Einstein, F-69621 Villeurbanne, France.

[2] Univ Lyon, INSA Lyon, Université Lyon 1, UMR 5240, CNRS, MAP, Microbiologie, Adaptation, Pathogénie, F-69621, Villeurbanne Cedex, France.

Supplementary data

* Corresponding authors at: ICBMS, Chimie Organique et Bioorganique, INSA-Lyon, 20 avenue Albert Einstein, Villeurbanne F-69621, France. Fax: +33 4 72 43 88 96.
E-mail address: Yves Queneau@insa-lyon.fr and laurent.soulere@insa-lyon.fr

^{1}H NMR Spectra

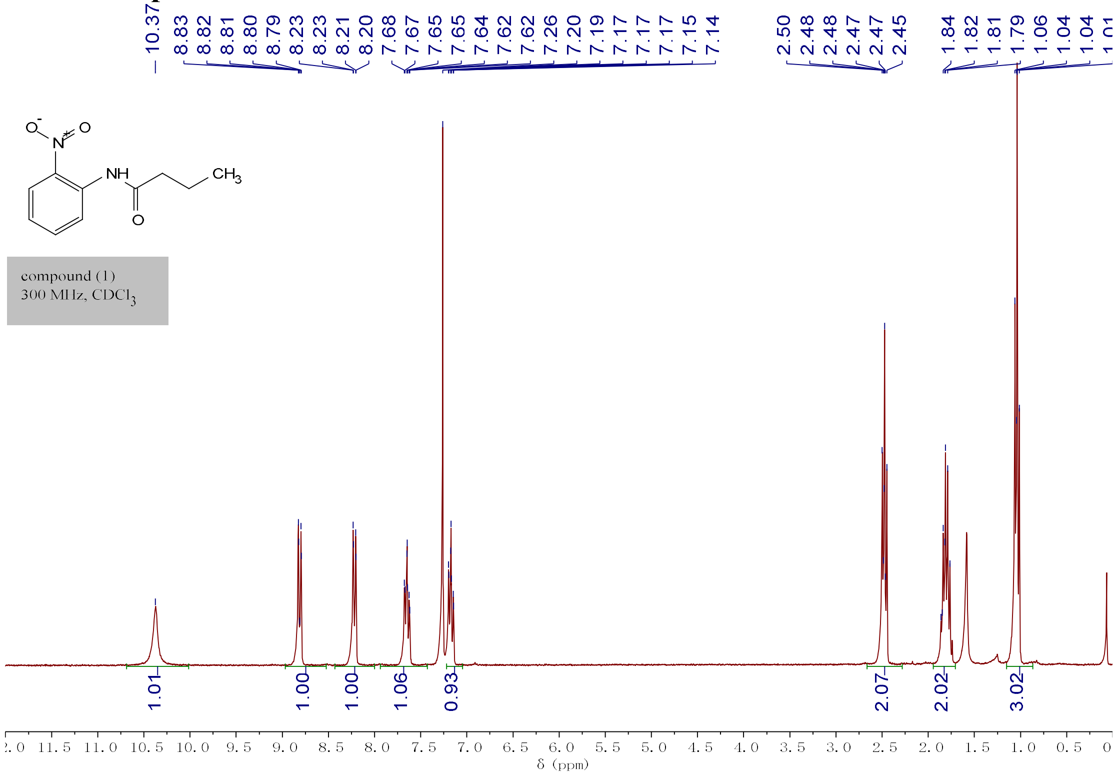

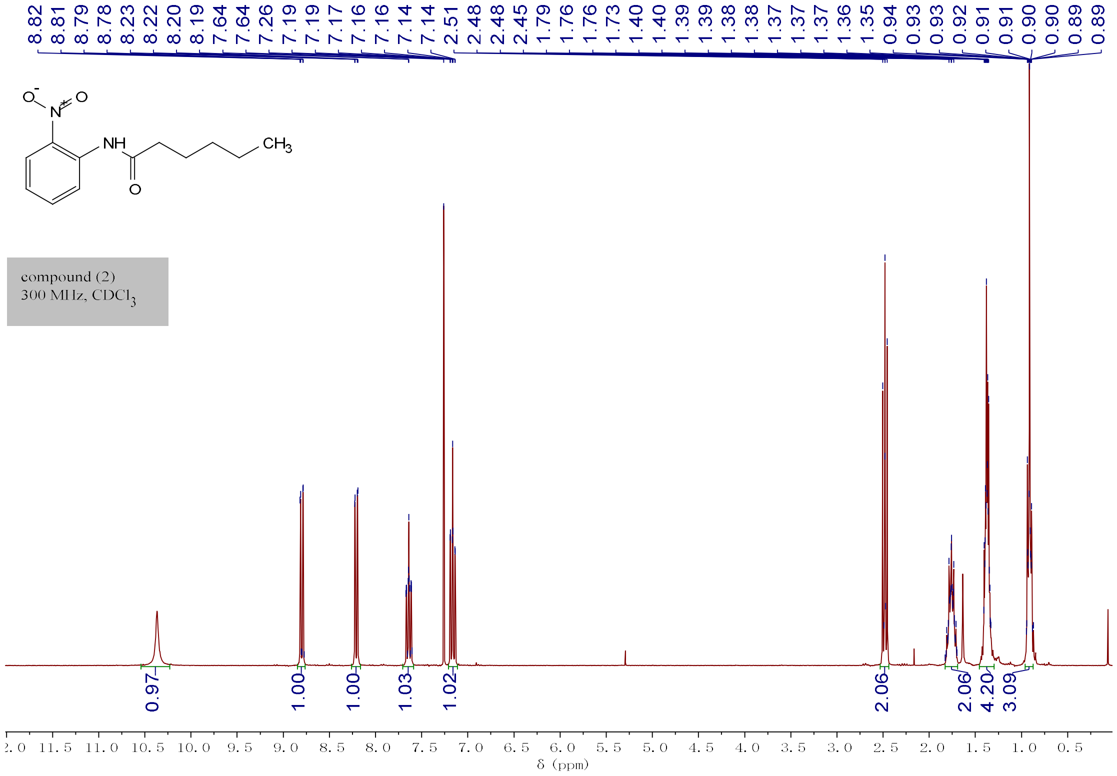

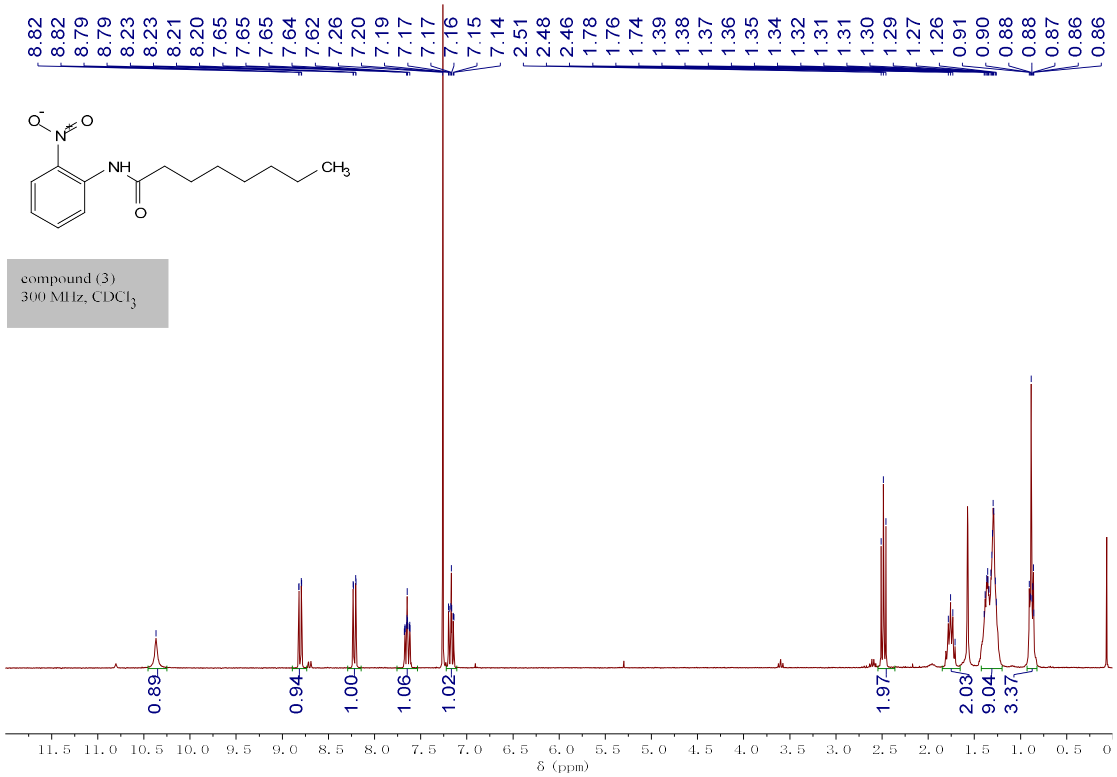
8.82 8.82 8.79 8.79 8.23 8.23 8.21 8.20 7.65 7.65 7.64 7.62 7.26 7.20 7.19 7.17 7.17 7.16 7.15 7.14 2.51 2.48 2.46 1.78 1.76 1.74 1.39 1.38 1.37 1.36 1.35 1.34 1.32 1.31 1.31 1.30 1.29 1.27 1.26 0.91 0.90 0.88 0.88 0.87 0.86 0.86
NH
CH3
compound (3)
300 MHz, CDCl3
0.89 0.94 1.00 1.06 1.02 1.97 2.03 9.04 3.37
δ (ppm)

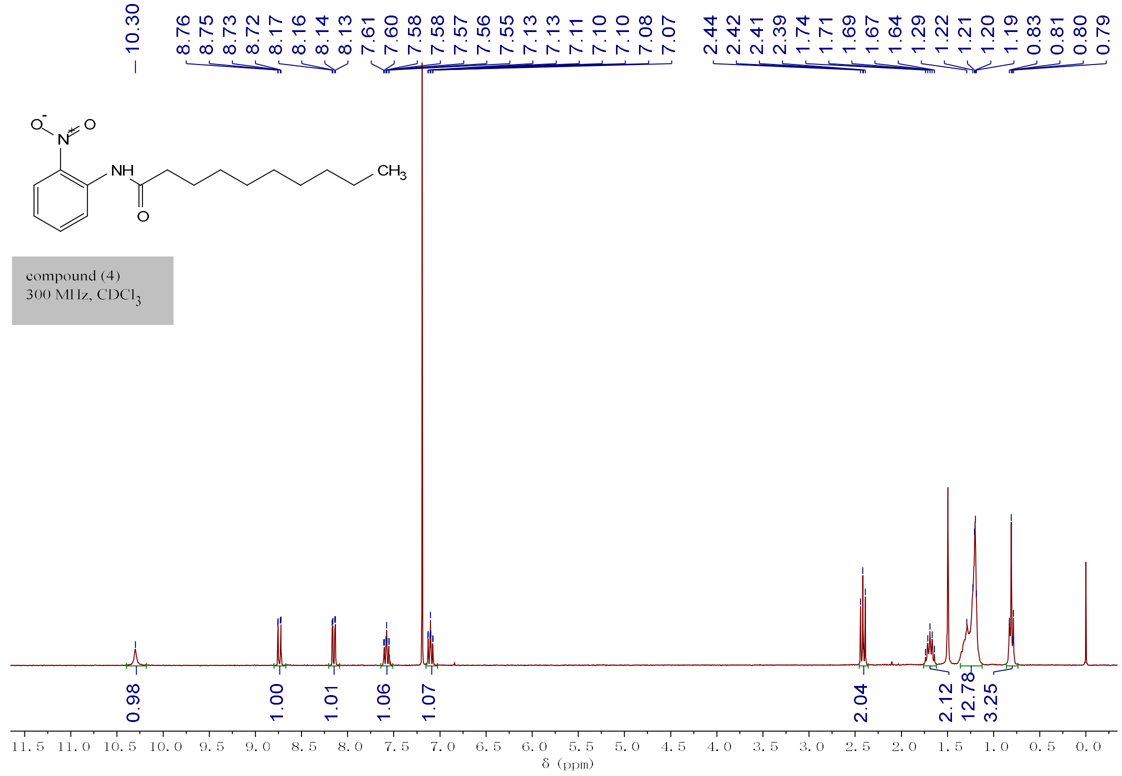
10.30
8.76 8.75 8.73 8.72 8.17 8.16 8.14 8.13 7.61 7.60 7.58 7.57 7.56 7.55 7.13 7.13 7.11 7.10 7.08 7.07 2.44 2.42 2.41 2.39 1.74 1.71 1.69 1.67 1.64 1.29 1.22 1.21 1.20 1.19 0.83 0.81 0.80 0.79
NH
CH3
compound (4)
300 MHz, CDCl3
0.98 1.00 1.01 1.06 1.07 2.04 2.12 12.78 3.25
δ (ppm)

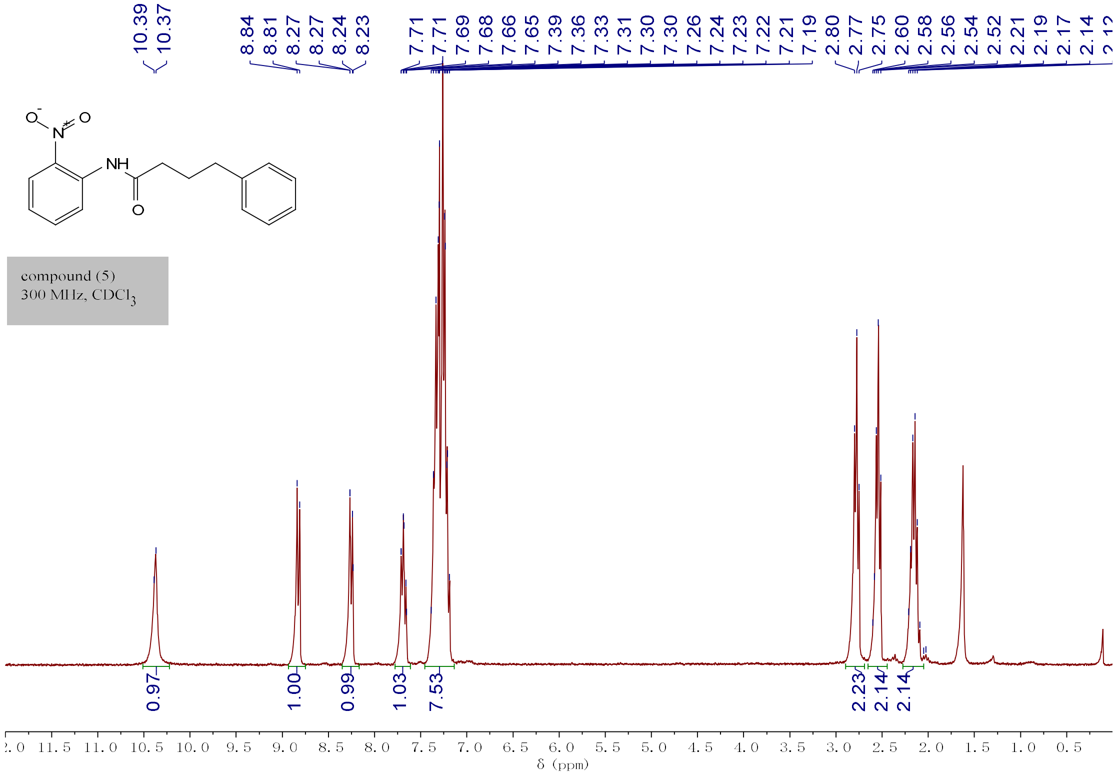
compound (5)
300 MHz, CDCl3
δ (ppm)

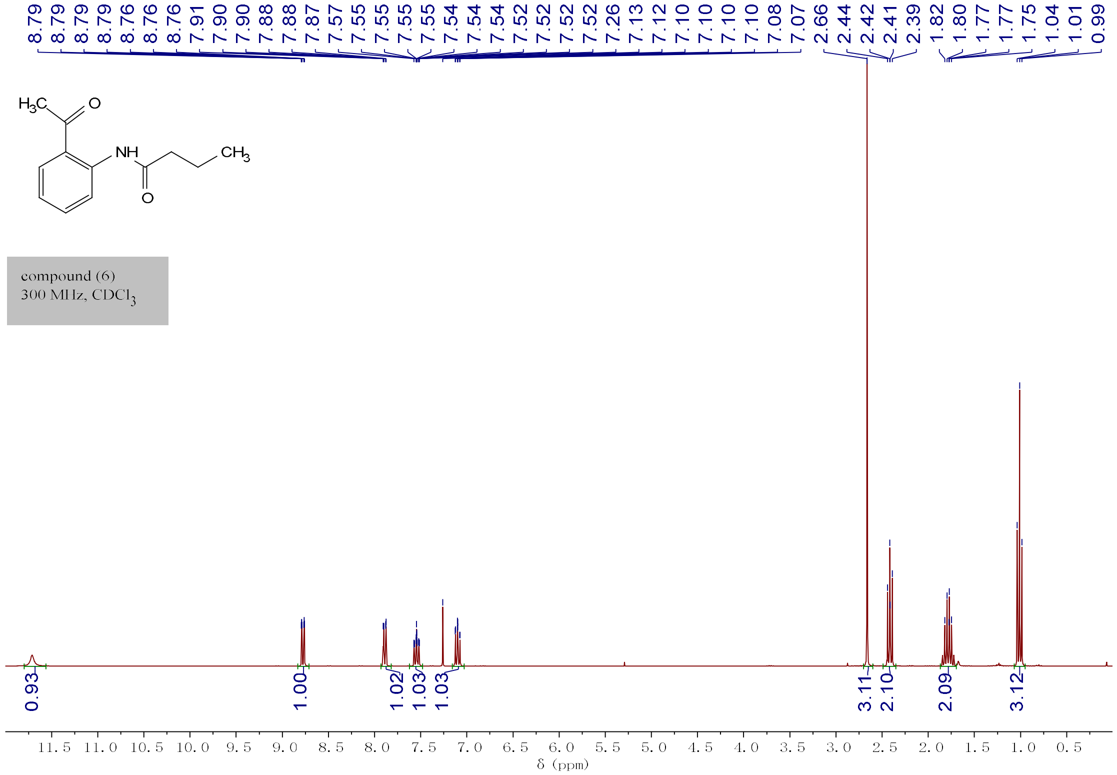
compound (6)
300 MHz, CDCl3
δ (ppm)

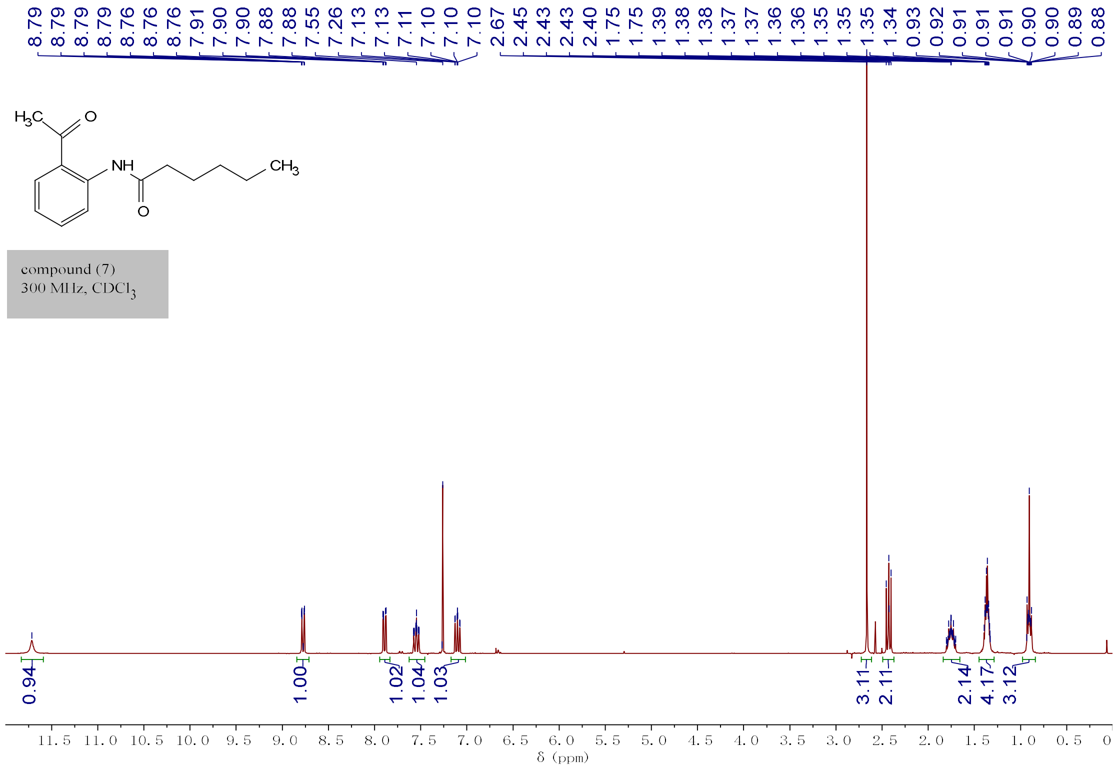
compound (7)
300 MHz, CDCl3
δ (ppm)

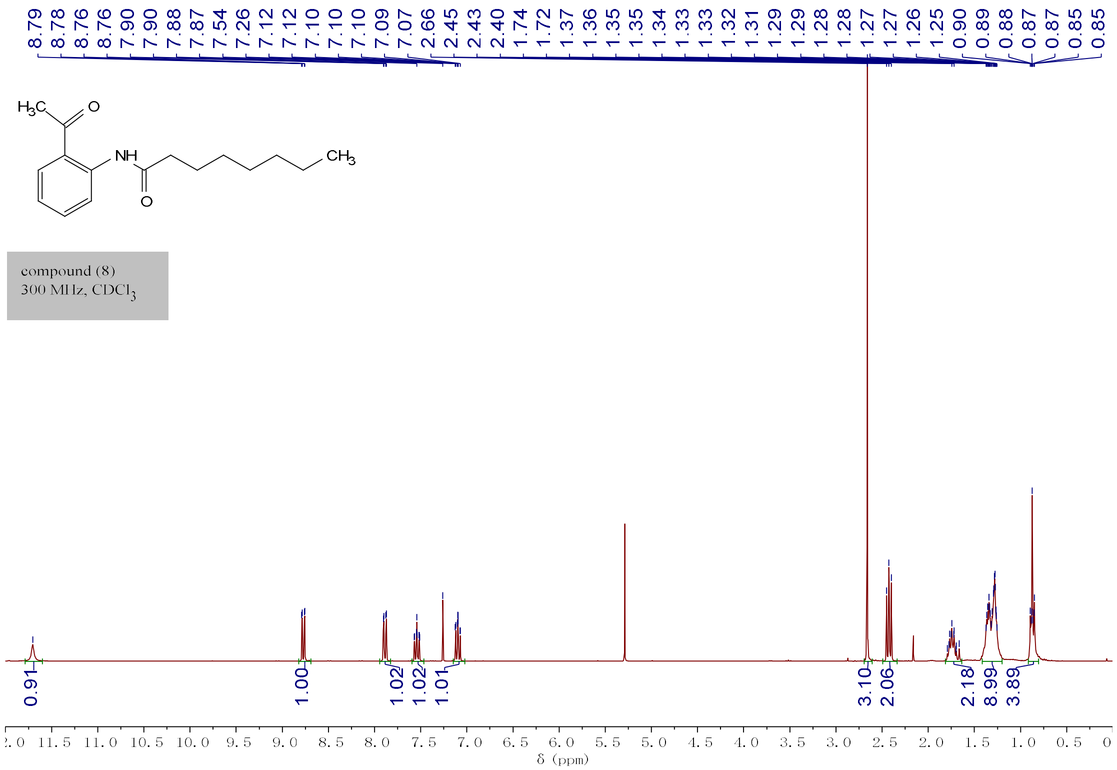
compound (8)
300 MHz, CDCl3
δ (ppm)

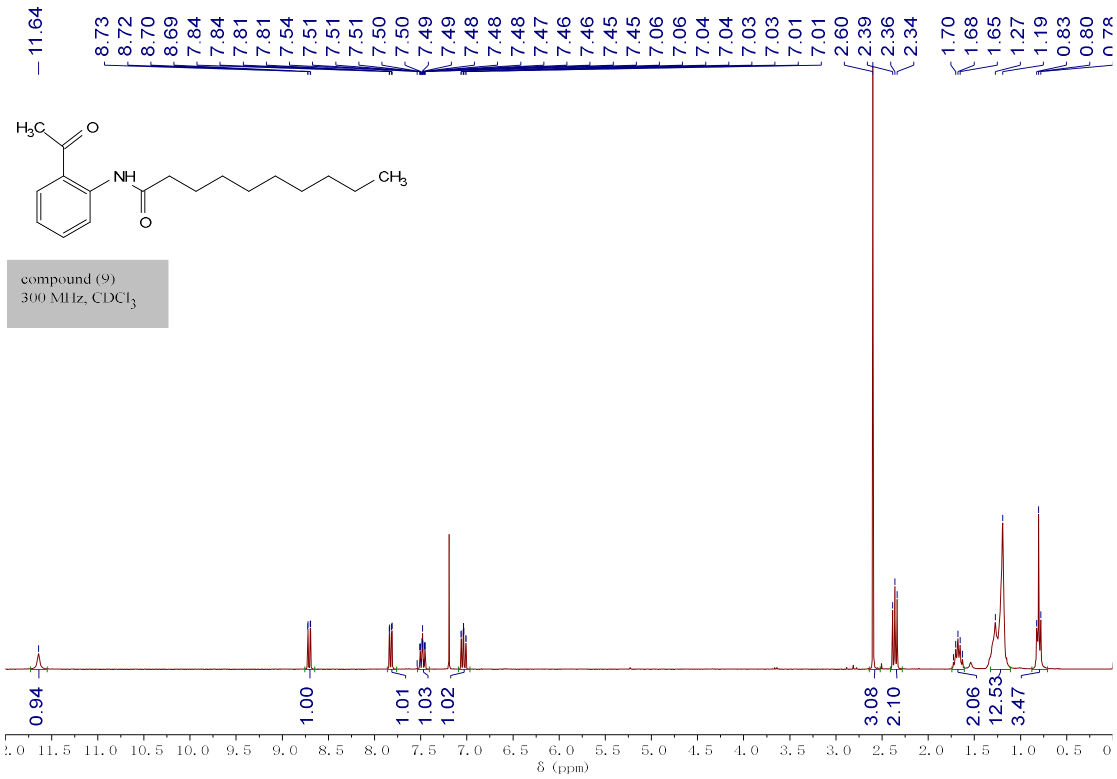
compound (9)
300 MHz, $CDCl_3$
δ (ppm)

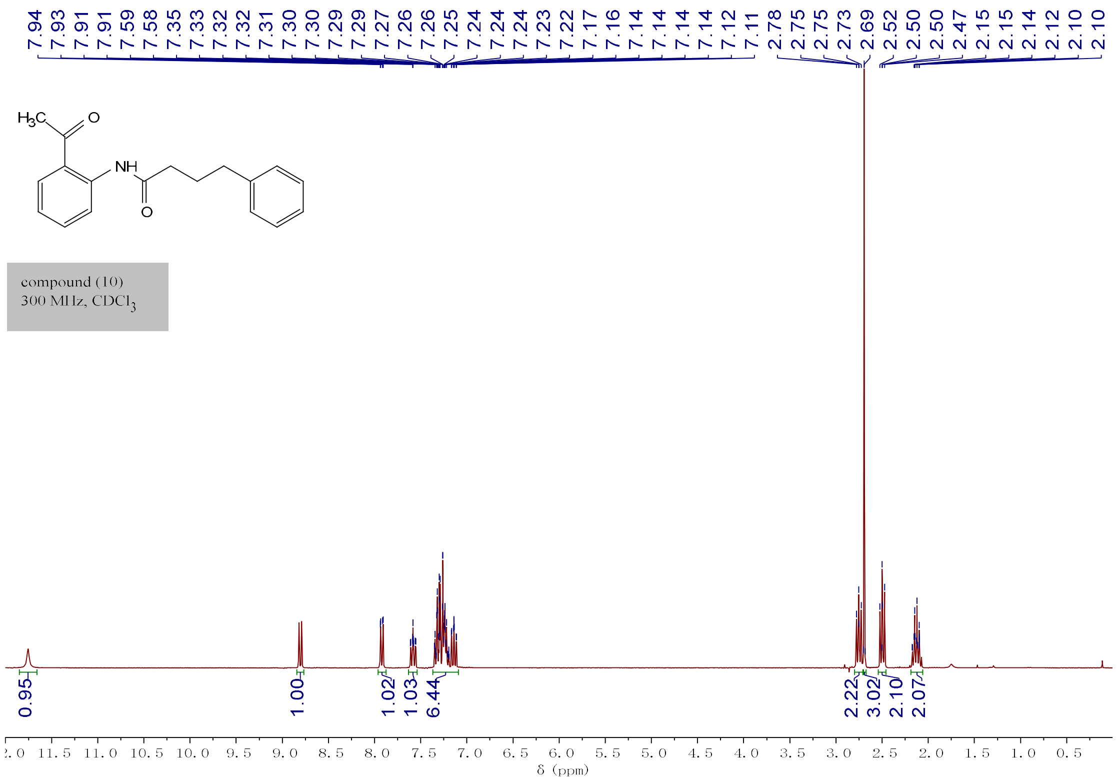
compound (10)
300 MHz, $CDCl_3$
δ (ppm)

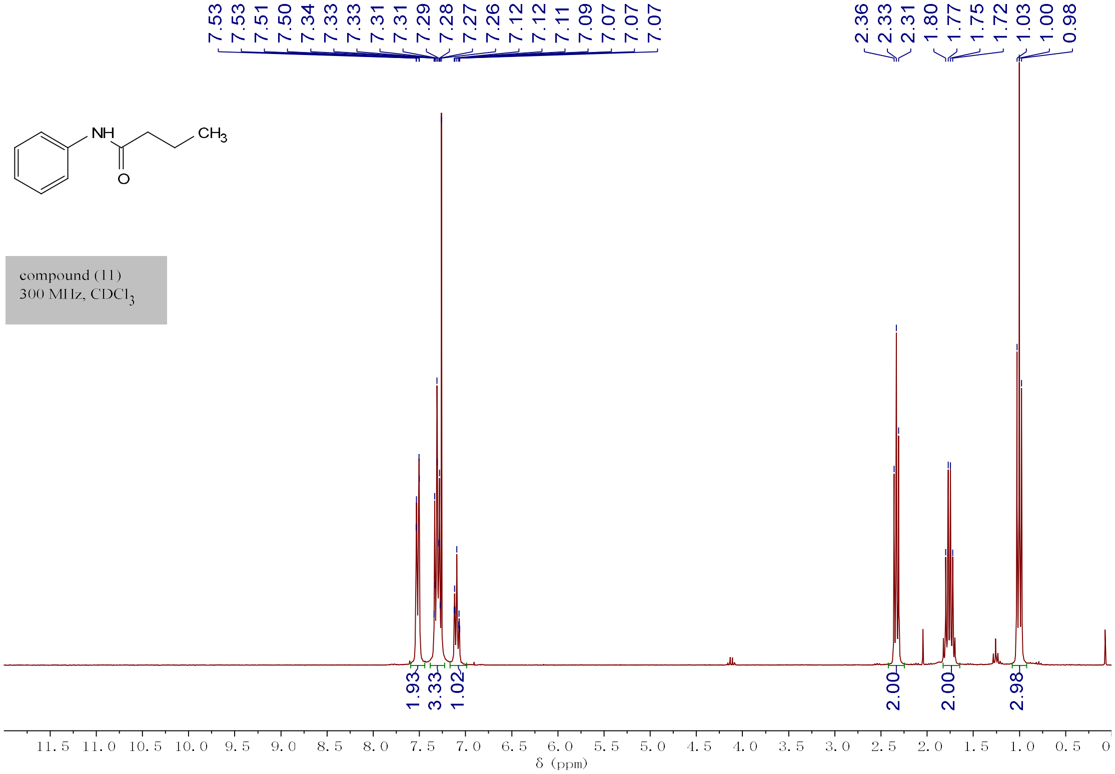
7.53 7.53 7.51 7.50 7.34 7.33 7.33 7.31 7.31 7.29 7.28 7.27 7.26 7.12 7.12 7.11 7.09 7.07 7.07 7.07
2.36 2.33 2.31 1.80 1.77 1.75 1.72 1.03 1.00 0.98
NH
CH3
O
compound (11)
300 MHz, CDCl3
1.93 3.33 1.02
2.00
2.00
2.98
11.5 11.0 10.5 10.0 9.5 9.0 8.5 8.0 7.5 7.0 6.5 6.0 5.5 5.0 4.5 4.0 3.5 3.0 2.5 2.0 1.5 1.0 0.5 0
δ (ppm)

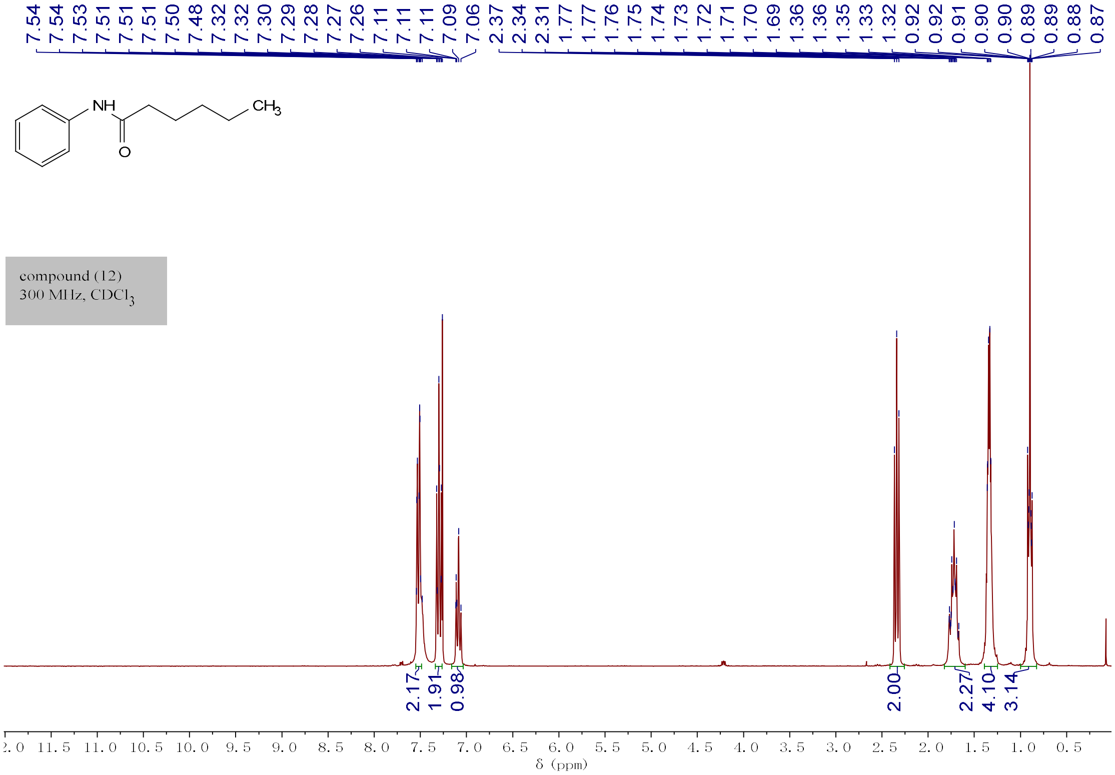
7.54 7.54 7.53 7.51 7.51 7.51 7.50 7.48 7.32 7.30 7.29 7.28 7.27 7.26 7.11 7.11 7.11 7.09 7.06
2.37 2.34 2.31 1.77 1.77 1.76 1.75 1.74 1.73 1.72 1.71 1.70 1.69 1.36 1.36 1.35 1.33 1.32 0.92 0.92 0.91 0.90 0.90 0.89 0.89 0.88 0.87
NH
CH3
O
compound (12)
300 MHz, CDCl3
2.17 1.91 0.98
2.00
2.27 4.10 3.14
12.0 11.5 11.0 10.5 10.0 9.5 9.0 8.5 8.0 7.5 7.0 6.5 6.0 5.5 5.0 4.5 4.0 3.5 3.0 2.5 2.0 1.5 1.0 0.5
δ (ppm)

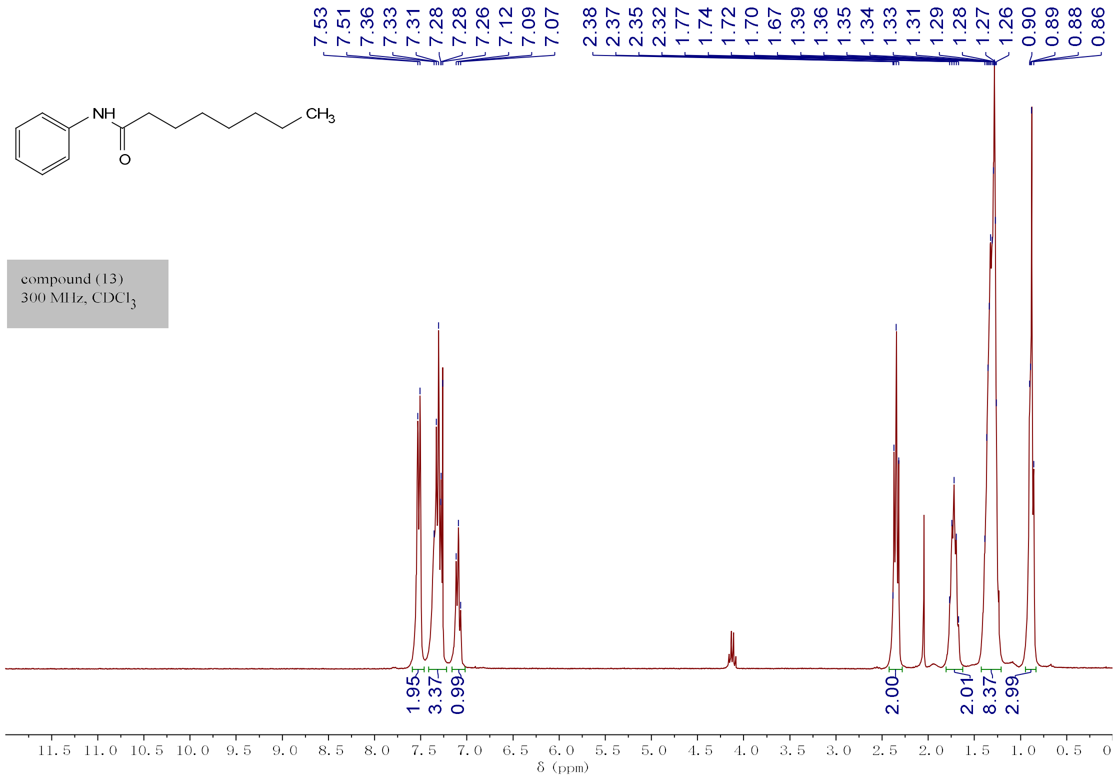
7.53 7.51 7.36 7.33 7.31 7.28 7.28 7.26 7.12 7.09 7.07
2.38 2.37 2.35 2.32 1.77 1.74 1.72 1.70 1.67 1.39 1.36 1.35 1.34 1.33 1.31 1.29 1.28 1.27 1.26 0.90 0.89 0.88 0.86
NH
O
CH_3
compound (13)
300 MHz, $CDCl_3$
1.95 3.37 0.99
2.00
2.01 8.37 2.99
δ (ppm)

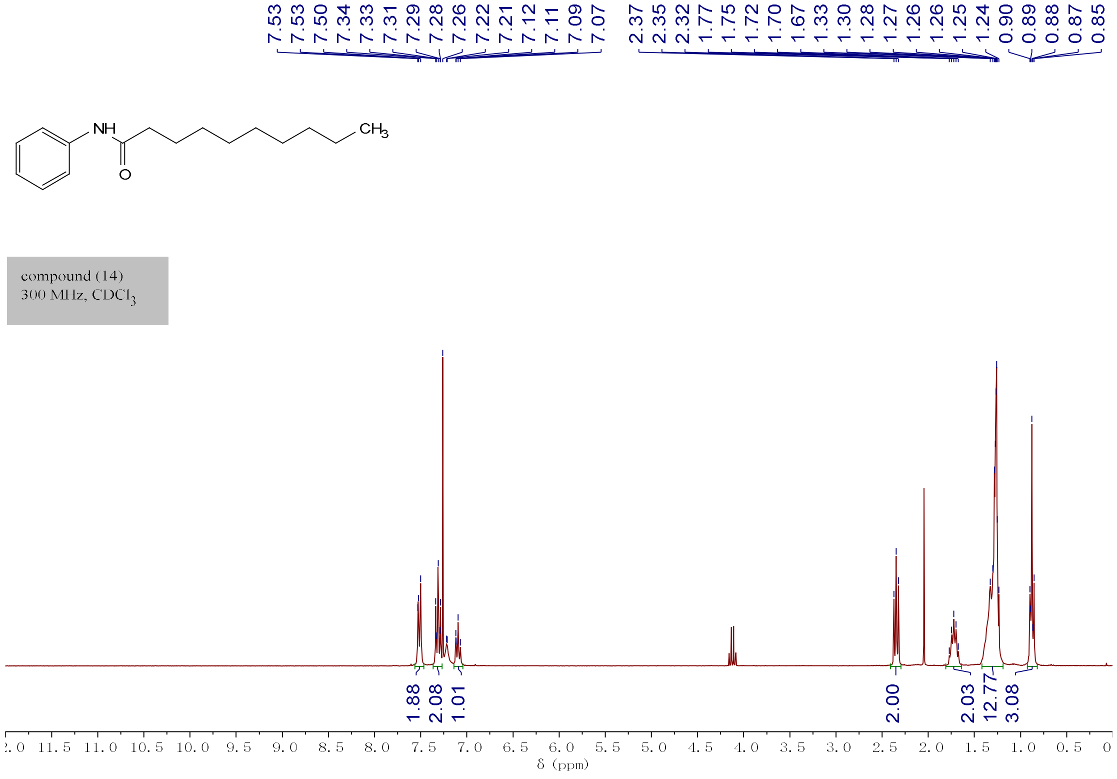
7.53 7.53 7.50 7.34 7.33 7.31 7.29 7.28 7.26 7.22 7.21 7.12 7.11 7.09 7.07
2.37 2.35 2.32 1.77 1.75 1.72 1.70 1.67 1.33 1.30 1.28 1.27 1.26 1.26 1.25 1.24 0.90 0.89 0.88 0.87 0.85
NH
O
CH_3
compound (14)
300 MHz, $CDCl_3$
1.88 2.08 1.01
2.00
2.03 12.77 3.08
δ (ppm)

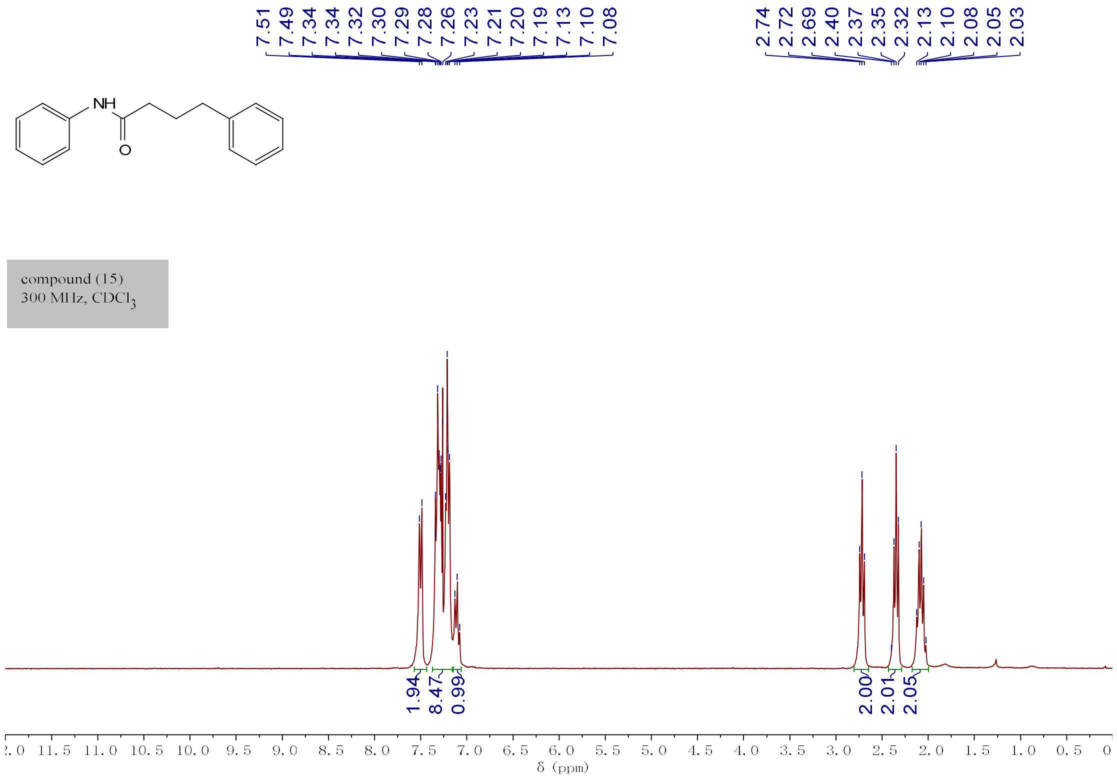
7.51 7.49 7.34 7.34 7.32 7.30 7.29 7.28 7.26 7.23 7.21 7.20 7.19 7.13 7.10 7.08
2.74 2.72 2.69 2.40 2.37 2.35 2.32 2.13 2.10 2.08 2.05 2.03
NH
O
compound (15)
300 MHz, $CDCl_3$
1.94 8.47 0.99
2.00 2.01 2.05
δ (ppm)
